# Amivantamab for Metastatic Lung Cancer With Paraneoplastic Disseminated Intravascular Coagulation: A Case Report

**DOI:** 10.7759/cureus.34033

**Published:** 2023-01-21

**Authors:** Yaser Ahmad, Tawee Tanvetyanon

**Affiliations:** 1 Medicine, Morsani College of Medicine, Tampa, USA; 2 Thoracic Oncology, H. Lee Moffitt Cancer Center, Tampa, USA

**Keywords:** disseminated intravascular coagulation, paraneoplastic syndrome, thrombocytopenia, lung cancer, amivantamab

## Abstract

Advanced cancers may be accompanied by paraneoplastic disseminated intravascular coagulation (DIC), resulting in thromboembolism or thrombocytopenia. Thrombocytopenia can limit the feasibility of myelosuppressive chemotherapy. Therefore, an alternative treatment option is needed. This report described a patient with metastatic pulmonary adenocarcinoma with *EGFR* exon 20 insertion and paraneoplastic DIC. Frontline chemotherapy failed to control the disease and worsened thrombocytopenia. However, treatment with amivantamab resulted in a rapid resolution of DIC and produced a partial tumor response. Based on our experience, for patients with *EGFR* exon 20 insertions with paraneoplastic DIC, amivantamab could be considered a preferred frontline treatment.

## Introduction

Disseminated intravascular coagulation (DIC) is a consumptive coagulopathy, which is triggered by abnormal activation of coagulation cascades. DIC can lead to platelet consumption and coagulation factor deficiency [1]. Certain solid tumors, including pulmonary adenocarcinoma, may release tumor-related procoagulants, giving rise to paraneoplastic DIC [2,3]. Clinical presentations of DIC may vary; however, common features include thromboembolism and thrombocytopenia [4]. In severe cases, bleeding may ensue. DIC in the context of malignancy is often compensated, not resulting in bleeding complications. Nonetheless, the condition can pose a therapeutic challenge due to thrombocytopenia, rendering myelosuppressive chemotherapy unsafe.

An activating mutation in the epidermal growth factor receptor gene (*EGFR*) may be present in over 15% of pulmonary adenocarcinomas [5]. This commonly occurs in exon 19 or exon 21 of the gene. Previous reports have indicated that tyrosine kinase inhibitor (TKI) targeting aberrant kinase can be effective for paraneoplastic DIC among patients with *EGFR* mutations [6]. However, to our knowledge, there has been no previous report of *EGFR* exon 20 insertions, an uncommon form of *EGFR* mutation, in association with paraneoplastic DIC. Although phenotypically similar to patients with common *EGFR* mutations, patients with the uncommon *EGFR* exon 20 mutations may have a poorer prognosis. In a study of 109 patients published in 2021, chemotherapy was found superior to conventional TKIs among patients with *EGFR* exon 20 insertions [7]. In this report, we described our experience treating a patient with *EGFR* exon 20 insertions with platinum-based chemotherapy, followed by amivantamab-a novel targeted therapy [8].

## Case presentation

**Figure 1:**
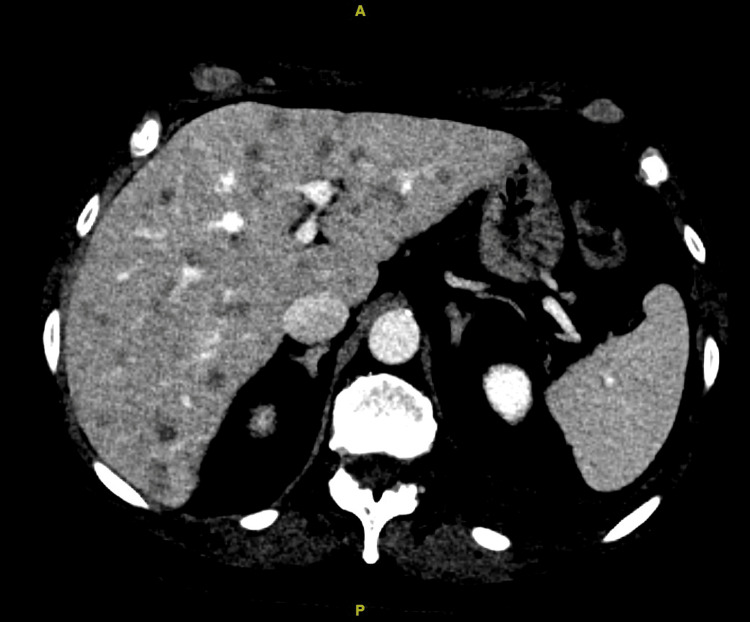
Pre-amivantamab computerized tomography of the abdomen revealing hepatic metastases

A 69-year-old female never-smoker presented with back pain and shortness of breath. She was found to have multiple lytic lesions in the spine. Computerized tomography (CT) of the chest and abdomen also showed lung masses along with multiple nodules in the liver (Figure 1).

A biopsy of the liver revealed pulmonary adenocarcinoma. Targeted next-generation sequencing of circulating tumor DNA was performed via Guardant360 (Guardant Health Incorporate, Redwood City, California), and this indicated *EGFR* exon 20 insertions (A767_V769dup) with 3.5% mutant allele fractions. The patient began treatment with palliative radiotherapy to the spine. Subsequently, she developed a pulmonary embolism and received apixaban.

She then presented for systemic therapy. The evaluation showed thrombocytopenia (52000/μL), increased D-dimer (20911 ng/mL) as well as decreased fibrinogen (141 mg/dL), consistent with DIC. She began chemotherapy with carboplatin and pemetrexed. Approximately one week later, her platelet count dropped to 9000/μL, necessitating a platelet transfusion. During this period, D-dimer and fibrinogen did not improve. A CT scan of the chest was performed, showing the progression of the disease.

**Figure 2:**
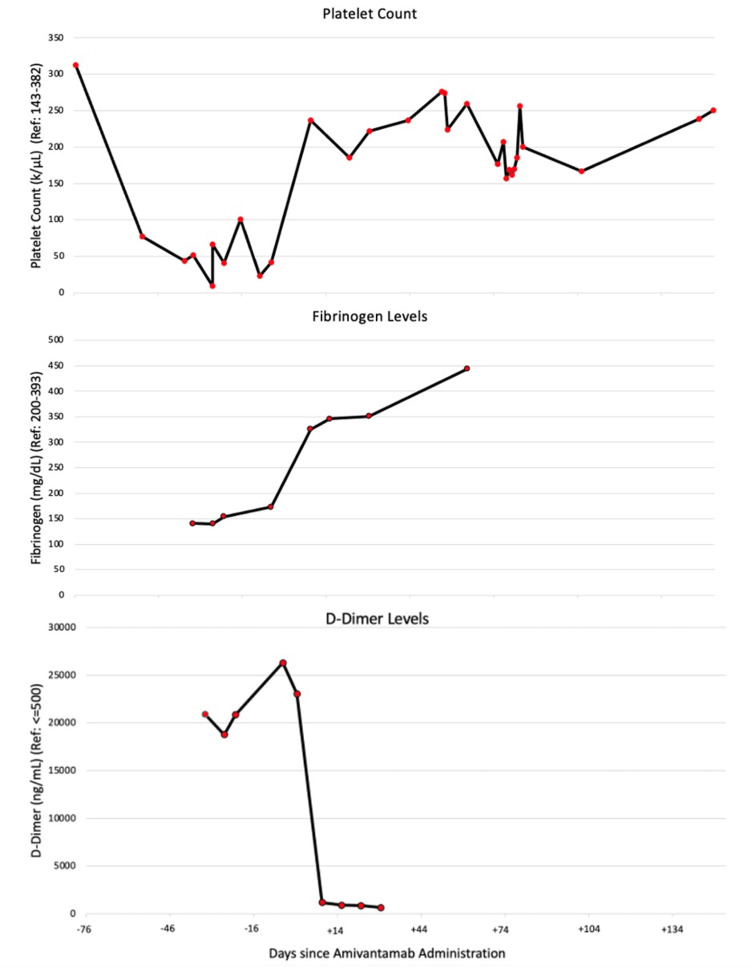
Platelet count, D-dimer, and fibrinogen level over time (day 0 representing the first amivantamab infusion)

Subsequently, the patient received amivantamab 1050 mg intravenously, administered weekly for four weeks, with the initial dose as a split infusion on the first and second days, every two weeks starting at the fifth week. She experienced mild infusion-related reactions during the first infusion, characterized by chills and myalgia. Over the course of the next two weeks, her platelet and D-dimer values became normal (Figure 2).

**Figure 3:**
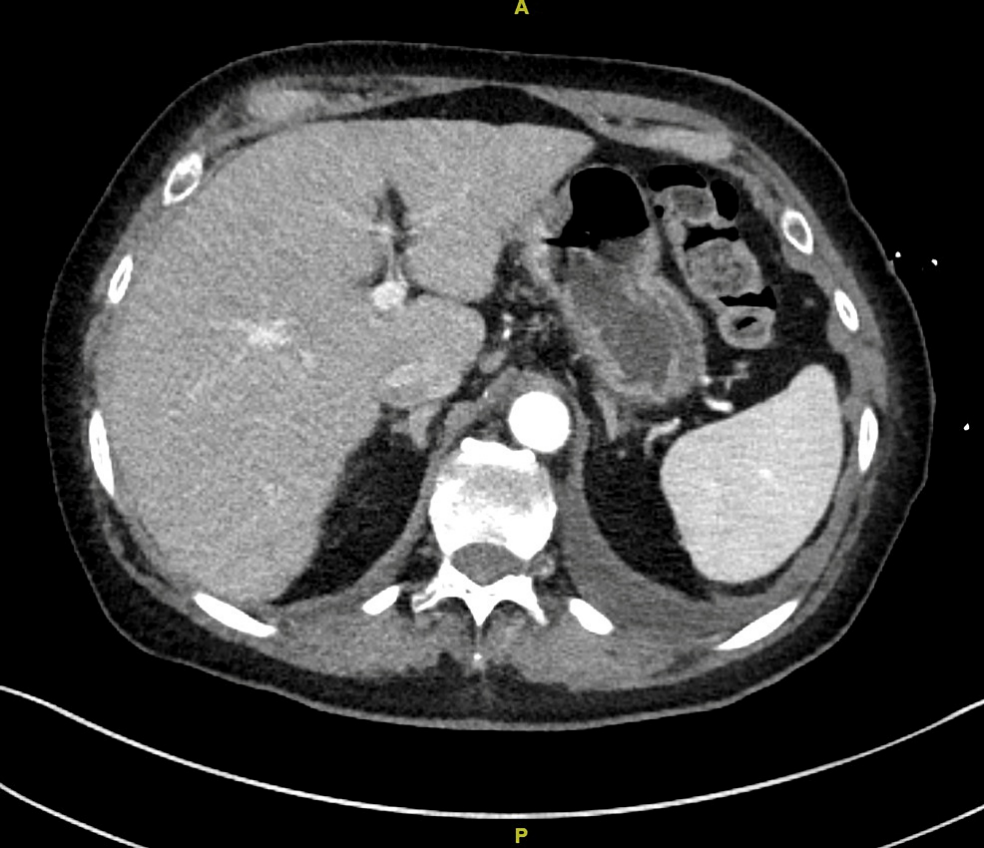
Post-amivantamab computerized tomography demonstrating a marked improvement in the hepatic metastases

Subsequently, a CT scan of the chest and abdomen demonstrated a partial tumor response (Figure 3).

The patient continued on amivantamab without any recurrence of DIC for approximately seven months afterward at the time of this report.

## Discussion

We described a patient with paraneoplastic DIC, occurring in the setting of metastatic pulmonary adenocarcinoma harboring *EGFR *exon 20 insertions. Chemotherapy failed to control the disease and worsened the DIC. However, amivantamab resulted in a prompt resolution of DIC and partial tumor response. To our knowledge, this is the first report of amivantamab treatment in the setting of paraneoplastic DIC.

Our findings are consistent with prior studies in that lung cancers harboring targetable *EGFR* mutation and paraneoplastic DIC respond well to appropriate targeted therapy. In a recent literature review of metastatic lung cancer patients with paraneoplastic DIC treated with targeted therapy, the one-year overall survival rate was estimated to be 69% [6]. With regard to the time to normalization of platelet count, some authors have reported an interval as short as three days after initiation of TKI [9]. Others have observed platelet count levels to become normal after one to two weeks following TKI initiation [10,11]. This appears consistent with our observed interval of two weeks. In one previous report, an early elevation of D-dimer was described, followed by a subsequent decrease [12]. However, we did not observe this pattern.

Prior to 2022, no targeted therapy was available for lung cancer patients with *EGFR* exon 20 insertions. At the time of this report, amivantamab is approved by the United States Food and Drugs Administration (FDA), only for subsequent lines of therapy [8]. The approval was based on data from a multi-cohort study of 81 patients who developed disease progression after platinum-based chemotherapy. In that study, amivantamab produced an overall response rate of 40% with a median response duration of 11 months [13]. The Food and Drug Administration (FDA) also approved Guardant360 as a companion diagnostic test. Amivantamab is distinct from other EGFR inhibitors in that it is administered intravenously, not orally. An immunoglobulin G (IgG)-based, bispecific antibody against EGFR and MET receptors, amivantamab not only results in signal inhibition but also mediates antibody-dependent cellular cytotoxicity, resulting in antibody-dependent phagocytosis [14]. Its common side effects include rashes and infusion-related reactions. Other serious, uncommon side effects may include pneumonitis and keratitis. It should be noted that mobocertinib represents another therapeutic option for *EGFR* exon 20 insertions. In a clinical trial, mobocertinib produced an overall tumor response rate of approximately 30% [15]. Since both amivantamab and mobocertinib do not affect hematopoiesis, we believe that they may be safer than chemotherapy as frontline therapy for patients with concurrent paraneoplastic DIC.

The strength of our report includes the availability of serial fibrinogen, D-dimer, and platelet count to characterize the time course relationship with amivantamab treatment. As a case report, however, we cannot directly compare between chemotherapy and amivantamab. Only data from a prospective study can fully characterize the safety and efficacy of frontline amivantamab in comparison with chemotherapy. Nonetheless, due to the thrombocytopenia and history of thromboembolism among patients with paraneoplastic DIC, it is unlikely that this patient population will be included in prospective clinical trials. Based on the superior efficacy and safety of amivantamab in our patient, amivantamab should be considered as frontline therapy. Finally, another limitation of our report is that we cannot rule out the possibility that DIC in our patient was caused by radiotherapy. Though unlikely, radiation exposure may cause tissue injury, resulting in thrombocytopenia [16].

## Conclusions

Amivantamab was safe and effective for our patient with *EGFR* exon 20 insertion and paraneoplastic DIC. We observed no bleeding complications during the treatment and the resolution of thrombocytopenia occurred just after two weeks of treatment. Although frontline treatment with amivantamab has not yet been approved by the FDA at the time of this report, we believe that amivantamab could be considered as a preferred frontline treatment for patients with *EGFR* exon 20 insertions with paraneoplastic DIC.
